# Spectral EEG correlations from the different phases of general anesthesia

**DOI:** 10.3389/fmed.2023.1009434

**Published:** 2023-03-06

**Authors:** Christophe Sun, Dan Longrois, David Holcman

**Affiliations:** ^1^Group of Data Modeling, Computational Biology and Predictive Medicine, Institut de Biologie (IBENS), École Normale Supérieure, Université PSL, Paris, France; ^2^Département d'Anesthésie-Réanimation, Hôpital Bichat-Claude Bernard, Assistance Publique-Hôpitaux de Paris, Paris, France; ^3^Churchill College, Cambridge, United Kingdom

**Keywords:** EEG segmentation, optimal fit, correlation analysis, time-frequency analysis, alpha rhythms, iso-electric suppressions, Bayesian statistics

## Abstract

**Introduction:**

Electroencephalography (EEG) signals contain transient oscillation patterns commonly used to classify brain states in responses to action, sleep, coma or anesthesia.

**Methods:**

Using a time-frequency analysis of the EEG, we search for possible causal correlations between the successive phases of general anesthesia. We hypothesize that it could be possible to anticipate recovery patterns from the induction or maintenance phases. For that goal, we track the maximum power of the α−band and follow its time course.

**Results and discussion:**

We quantify the frequency shift of the α−band during the recovery phase and the associated duration. Using Pearson coefficient and Bayes factor, we report non-significant linear correlation between the α−band frequency and duration shifts during recovery and the presence of the δ or the α rhythms during the maintenance phase. We also found no correlations between the α−band emergence trajectory and the total duration of the flat EEG epochs (iso-electric suppressions) induced by a propofol bolus injected during induction. Finally, we quantify the instability of the α−band using the mathematical total variation that measures possible deviations from a flat line. To conclude, the present correlative analysis shows that EEG dynamics extracted from the initial and maintenance phases of general anesthesia cannot anticipate both the emergence trajectory and the extubation time.

## 1. Introduction

Time-frequency analysis (1, 2) applied to the electroencephalography (EEG) signal has revealed persistent and transient oscillatory bands that are used to classify the brain states (3). Often used to quantify the different oscillatory bands, signal processing parameters include the power spectrum (4), the energy of each band (2), but also the duration of iso-electric suppressions (IES) or α−band suppressions (5), that are segments where the amplitude is below a given threshold. These quantifications benefited from wavelet approaches (6–9) to filter the EEG in real time or to remove noisy perturbations or artifacts (10, 11).

The paradigm shift from EEG correlative and statistical analysis to a predictive approach has relied on the maturation of machine learning approaches (12). Thus, predicting brain sensitivity during general Anesthesia (GA) became possible by quantifying the abundance of IES, markers of a deep sedation or a “vulnerable brain” (13).

These local flat EEG events could be anticipated by the dynamics of α−band suppressions (5), or by combining first passage events, such as the first occurrence time when IES appeared (14). Low frontal α power was also found to be associated with an increase probability of finding IES and Burst-Suppression in the EEG signal (13, 15). These results called for more correlations between the three stages of GA consisting in (1) induction, (2) maintenance, and (3) recovery.

First, the induction phase recapitulates the beginning of hypnotic drug administration, such as propofol or sevoflurane (16–18). These drugs create an artificial and reversible coma preventing brain memorization of surgical events. Induction is the transition period from an awake to a sedative state. The initial hypnotic dosage is often calibrated on patient age and is proportional to the weight (19). This phase lasts from few to around 10 min, as long as necessary to adjust the patient into a stable sedative state. Induction is also associated with a change of the EEG spectral frequency bands with the appearance of a stable α−oscillation in the 8–12 Hz frequency band characterizing this loss of consciousness (LOC). Second, during the maintenance phase, the anesthetic dosage is often adjusted on monitoring machines to maintain the EEG frequencies with the presence of δ and α rhythms during the entire surgical procedure. However, the α−band can sometimes disappear and periods of IES and Burst-Suppression could accumulate over time. It remains an open question to anticipate the brain propensity to generate these EEG instabilities from the induction phase, that could be interpreted as a sign of excessive anesthesia depth. Finally, the recovery phase is associated to the return to consciousness once the anesthetic dosage is gradually cut off. The EEG spectrogram shows a shift toward higher frequency bands (β and γ) and the spectral analysis reveals a stereotype “zip”-shape signature, where the maximum power frequency within the α−band increases during the recovery of consciousness (ROC), while the δ−band decreases until it disappears (20, 21). However, the EEG signal during recovery is not the reversal of the one recorded during induction, as it is controlled by neuronal circuit recovery due to hypnotic elimination, while the time scale of LOC is associated to dominance of cortical inhibitory neuronal synchronization (22–24).

Previous works (5, 14) showed that the statistics of αS during induction correlates with the appearance of IES during maintenance. We explore in the present manuscript, whether other correlations could exist between the three phases of GA. For example, a sensitivity detected during induction could lead to many IES and thus overdosage, leading ultimately to a possible longer time for ROC. Finding further correlations requires to analyze the dynamics of the α−band by computing mathematical indices such as the total variation *V*_α_ of the maximum α−oscillations (2). This total variation measures the deviation of α−oscillations maximum amplitude from a flat line. We will develop a fitting procedure for this band and estimate the duration of the α−band emergence trajectory, during which the frequency shifts significantly upwards after the hypnotic injection is stopped. We use these parameters to study the possible correlations between the recovery and the previous phases of anesthesia. We quantify the duration and frequency shifts of the α−band emergence trajectory for the ROC by fitting the power of the α−band with a sigmoid function. Finally, exploring whether there are correlations in the EEG signal between the three phases of GA could be used to anticipate the next phase from the previous ones. We report here mostly weak correlations between the various indices. These results suggest that the sedation depth of the brain should be constantly monitored toward an optimal state: neither too deep nor too light, which remains to be formulated in mathematical terms.

## 2. Materials and methods

### 2.1. EEG recordings and preprocessing

EEG monitoring was performed by the Masimo Root^®^ monitor with four frontal electrodes F7, Fp1, Fp2, F8 in the 10–20 system. We segmented motion-induced mechanical noise using the EEG signal power within a sliding window of length 10 s and 50% overlap. When the signal power within a window *P*_*i*_ exceeds three times the median absolute value


(1) $$\begin{array}{l} MAD=\text{median}\left(|P_{i}-\text{median}\left(P\right)|\right), \end{array}$$

For *i* = 1, 2,.., *N*, where *N* is the number of windows, we label this event as an artifact. The artifact segments are then corrected using the Wavelet Quantile Normalization algorithm (10, 11).

### 2.2. Quantification of the α−band emergence trajectory

**Figure 1:**
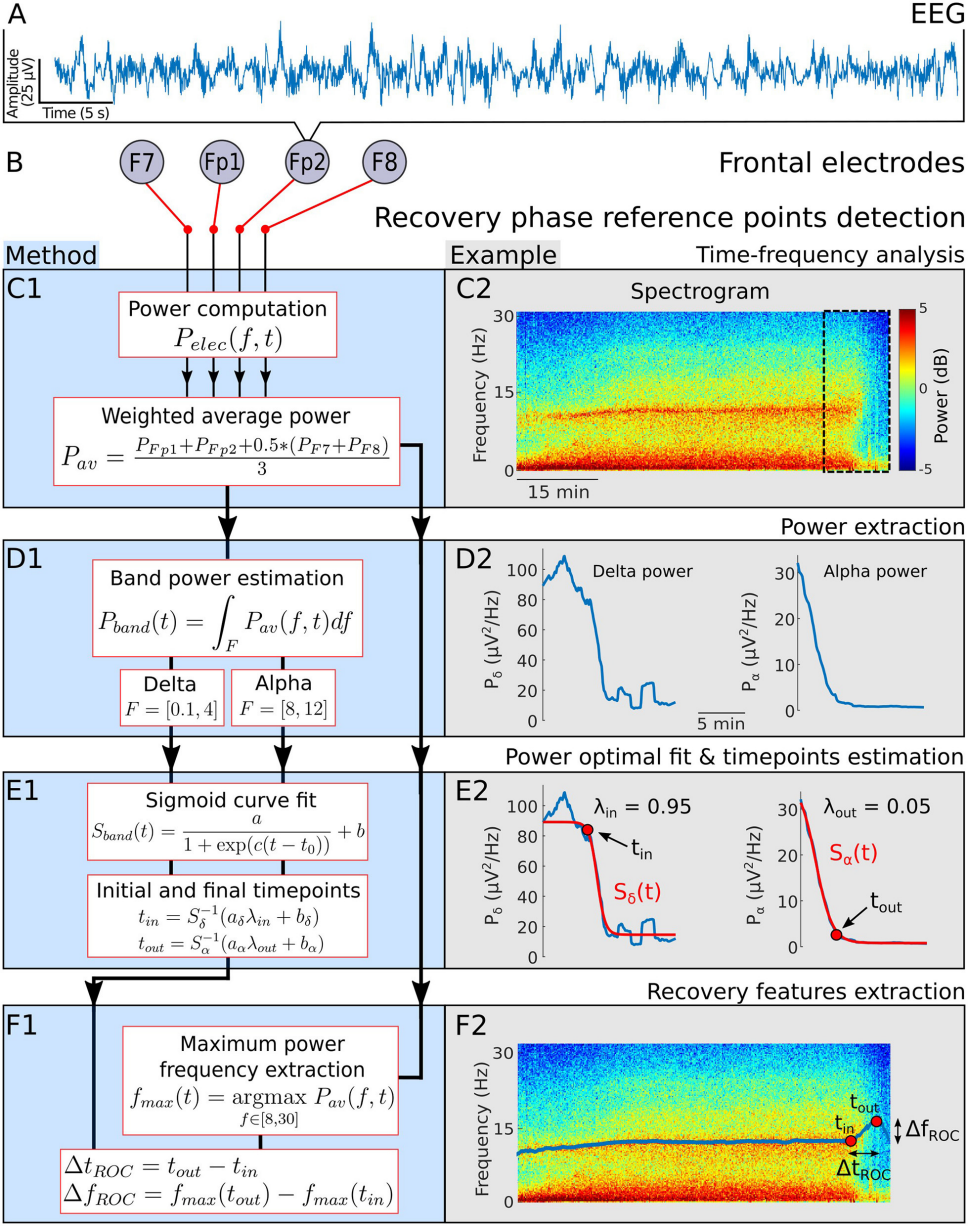
Emergence phase detection. **(A)** EEG signal. **(B)** Frontal electrodes. **(C) 1-** Power of a weighted average of the four EEG channels. **2-** Spectrogram visualization for a single patient during general anesthesia. **(D) 1-**α−band power *P*_α_ and δ−band power *P*_δ_ **2-** Power *P*_α_ and *P*_δ_ computed from the spectrogram 20 min before the end of anesthesia. **(E) 1-** Sigmoid function *S*_*band*_ used to fit *P*_α_ and *P*_δ_ during the recovery phase. Definition of the final time *t*_*in*_ (resp. *t*_*out*_) for which the fit *S*_δ_ (resp. *S*_α_) reaches for the first time the threshold *aλ*_*in*_+*b* (resp. *aλ*_*out*_+*b*). **2-** Fit examples and detection of *t*_*in*_ and *t*_*out*_. **(F) 1-** Maximum power frequency *f*_*max*_ and definition of the duration and frequency shifts during recovery. **2-** Extracted *f*_*max*_ and positioned *t*_*in*_ and *t*_*out*_ on the associated spectrogram.

To quantify the emergence trajectory of the α−band from the EEG, we introduced two reference time points *t*_*in*_ and *t*_*out*_ to characterize the beginning and the end for the EEG change during the recovery phase (Figure 1). The detection procedure of the these time points started with applying a Fourier transform on a 20 s sliding window and 75% overlap to obtain the spectrogram of the EEG signal from the four frontal electrodes *P*_*Fp*1_, *P*_*Fp*2_, *P*_*F*7_ and *P*_*F*8_ (Figures 1A–C).

**Figure 2:**
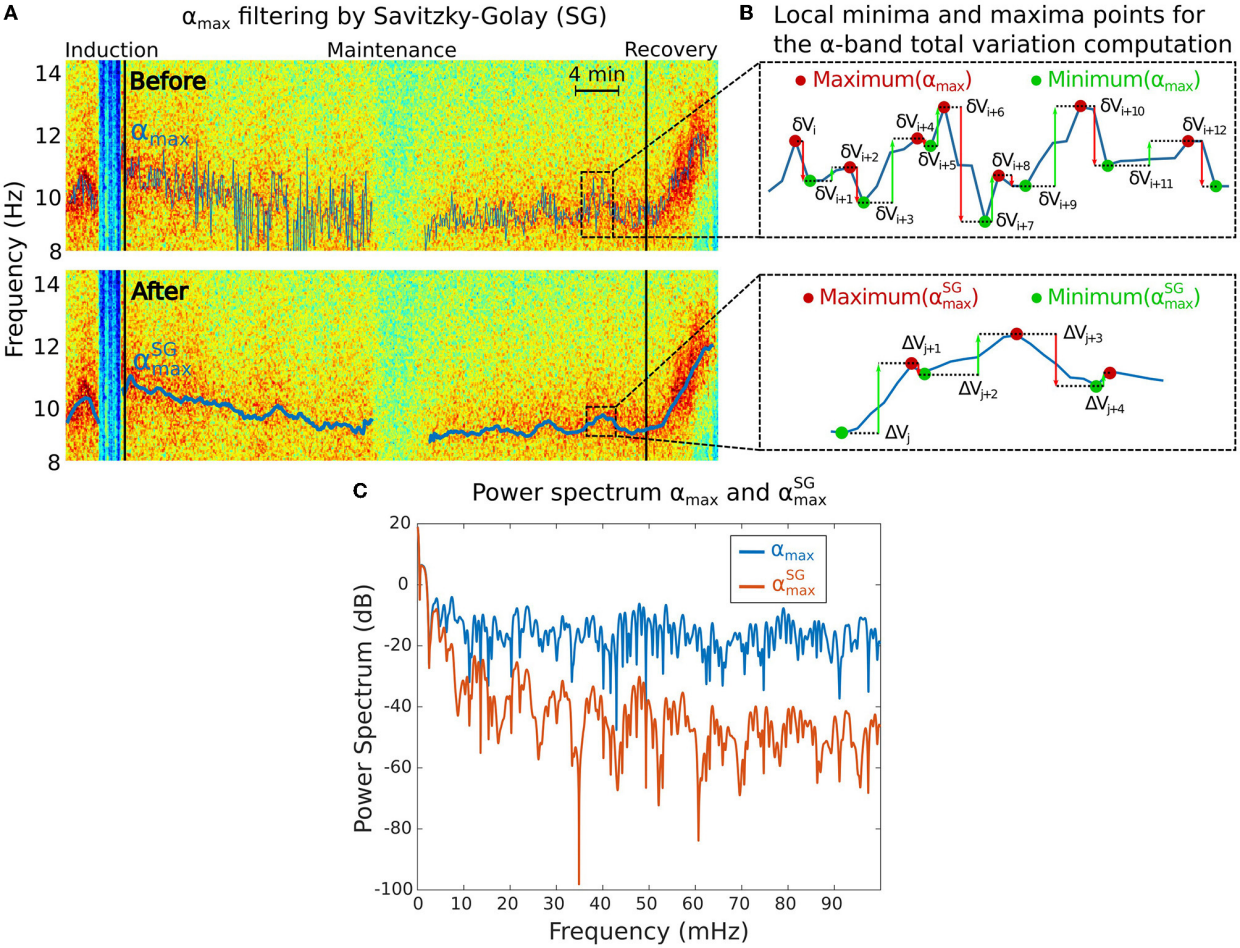
Total variation *V*_α_ analysis associated to the α−band. **(A)** Maximum power frequency α_*max*_ of the α−oscillations before (top) and after (bottom) applying the Savitzky-Golay (SG) filter to remove the high frequencies. **(B)** Reduction of the local minima and maxima variations of the α_*max*_ from the initial signal (top) to the filtered one (bottom). **(C)** Power spectra of α_*max*_ before (blue) and after (red) SG filtering showing that only the slowest oscillations remain in the filtered signal.

To account for the predominant role of the frontal cortex in the genesis of the α−band during GA (25, 26), we averaged the spectrograms with different weights: we halved the contribution of electrodes F7 and F8 (Figures 1, 2). We then followed the α−band power *P*_α_ and δ−band power *P*_δ_. The signal power within a band *P*_*band*_(*t*) is equal to the area under the curve *P*_*av*_(*f, t*), where the frequency *f* varies in a given band, as shown in Figures 1, 2. Specifically,


(2) $$P_{\delta }(t)=\int_{f\in [0.1,4]}P_{av}(f,t)\text{d}f.$$

(3) $$P_{\alpha }(t)=\int_{f\in [8,12]}P_{av}(f,t)\text{d}f.$$

We fitted separately the powers *P*_δ_, *P*_α_ with a sigmoid function


(4) $$\begin{array}{l} S_{k}\left(t\right)=\frac{a}{1+\exp\left(c\left(t-t_{0}\right)\right)}+b, \end{array}$$

where *k* = δ or α) (Figures 1, 2). The parameters *a, b, c* and *t*_0_ are estimated by minimizing error integral


(5) $$\{\hat{a},\hat{b},\hat{c},\hat{t_{0}}\}=\;\underset{a,b,c,t_{0}}{\arg\;\min}\int_{T_{f}-20}^{T_{f}}|S_{band}(s)-P_{band}(s){|}^{2}\text{d}s,$$

Where *T*_*f*_ is the last time where the EEG recordings terminates. The time point *t*_*in*_ is defined as the first time where the sigmoid curve *S*_δ_ crosses the threshold $y=â\lambda_{in}+\hat{b}$ with λ_*in*_ = 0.05. Equivalently, $S_{\delta }\left(t_{in}\right)=â\lambda_{in}+\hat{b}$, thus


(6) $$\begin{array}{l} t_{in}=\frac{1}{ĉ}\ln\left(\frac{1}{\lambda_{in}}-1\right)+\hat{t_{0}}. \end{array}$$

We also determined the frequency curve *f*_*max*_ with maximal power in the range (8-30) Hz (Figures 1, 2) using


(7) $$\begin{array}{l} f_{max}\left(t\right)=\underset{f\in \left[8,30\right]}{\mathrm{argmax}}P_{av}\left(f,t\right). \end{array}$$

Similarly, we define the time point *t*_*out*_ as the first time where *S*_δ_ crosses $y=â\lambda_{out}+\hat{b}$ with λ_*out*_ = 0.95, thus


(8) $$\begin{array}{l} t_{out}=\frac{1}{ĉ}\ln\left(\frac{1}{\lambda_{out}}-1\right)+\hat{t_{0}}. \end{array}$$

Using these two reference points *t*_*in*_ and *t*_*out*_, we deduced the duration and the frequency shifts associated with the α−band emergence trajectory (Figures 1, 2)


(9) $$\Delta t_{ROC}=t_{out}-t_{in}$$

(10) $$\Delta f_{ROC}=f_{max}(t_{out})-f_{max}(t_{in}).$$

### 2.3. Segmenting IES and α suppressions

The IES segmentation procedure follows the steps presented in Sun and Holcman (14) that we briefly recall. We first filter the EEG signal *S*(*t*) in the range [8,16] Hz to get *S*_α_(*t*). We normalize *S*_α_(*t*) by its Root-Mean-Square to obtain Ŝ_α_(*t*). We then estimated the upper and lower envelops of *S*(*t*) and Ŝ_α_(*t*) by interpolating their local maxima and minima. We applied the difference *D*(*t*) and *D*_α_(*t*) between the respective upper and lower envelops, and defines two threshold values for the IES:


(11) $$T_{IES}\;=\text{ min}(8,\text{ median}(D(t)))$$

(12) $$T_{\alpha }\;=\text{ min}(0.25,\text{ median}(D_{\alpha }(t))).$$

We then define the IES (resp. α*S*) time segment as the ensemble satisfying the conditions


(13) $$\begin{array}{lll} \Omega_{IES} & = & \left\{t\text{such that}|S\left(t\right)|<T_{IES}and|{Ŝ}_{\alpha }\left(t\right)|<T_{\alpha }\right\}. \end{array}$$

(14) $$\begin{array}{lll} \Omega_{\alpha S} & = & \left\{t\text{such that}|S\left(t\right)|>T_{IES}and|{Ŝ}_{\alpha }|<T_{\alpha S}\right\}. \end{array}$$

Finally, we aggregate the time intervals of Ω_*IES*_ and Ω_α*S*_ into segments such that each pair of consecutive points of the segment matches with the known sampling frequency. To smooth the effect of the hard thresholding, we used morphological erosion and dilation operations (27) to obtain the IES and αS duration intervals.

### 2.4. Mathematical indicators associated to iso-electric suppressions, α and δ bands

- The total time spent in IES is the sum of time segments during induction in Ω_*IES*_. For each *i*^*th*^ segment *T*_*i*_ which starts (resp. ends) at time *T*_*i, start*_ (resp. *T*_*i, end*_), we defined

(15) $$\begin{array}{l} S_{IES}=\underset{\left\{T_{i}\in \Omega_{IES}\right\}}{\sum }|T_{i,end}-T_{i,start}|. \end{array}$$

- The longest IES event is equal to the maximum of the duration present in Ω_*IES*_ during induction.

(16) $$\begin{array}{l} L_{IES}=\underset{T_{i}\in \Omega_{IES}}{\max}|T_{i,end}-T_{i,start}|. \end{array}$$

- The α−band relative power *P*_α_(*t*) at time t describes the proportion of energy in the range [8, 12] Hz with respect to [0.1, 45] Hz, as defined by

(17) $$P_{\alpha }(t)=\frac{\int_{f\in [8,12]}P_{av}(f,t)\text{d}f}{\int_{f\in [0.1,45]}P_{av}(f,t)\text{d}f}.$$

To compute the mean value of the relative α−band power ${\bar{P}}_{\alpha }$ during maintenance, we use the discretized sum approximation


(18) $$\begin{array}{l} {\bar{P}}_{\alpha }=\frac{1}{n+1}\overset{n}{\underset{i=0}{\sum }}P_{\alpha }\left(t_{i}\right). \end{array}$$

- The mean value of the δ−band relative power ${\bar{P}}_{\delta }$ is obtained following the procedure of *P*_δ_.
- The maximum power frequency α_*max*_ within the α− band is the frequency for which the power is maximal and defined outside of IES or αS regions.

(19) $$\begin{array}{l} \alpha_{max}\left(t\right)=f_{\alpha }\left(t\right)\cdot 1_{\bar{\Omega_{IES}\cup \Omega_{\alpha S}}}\left(t\right), \end{array}$$

where


(20) $$\begin{array}{l} f_{\alpha }\left(t\right)=\underset{f\in \left[8,12\right]}{arg max}P_{av}\left(f,t\right). \end{array}$$

The maximum frequency α_*max*_(*t*) contains multi-scale oscillations that do not necessarily reflect physiological information. We thus decided to smooth α_*max*_(*t*) using the adaptive Savitzky-Golay smoothing filter (28) to keep lower frequencies (Figure 2A). We used a sliding window of 2 min and 5 s step size with order 1 polynomial regression. We denote $\alpha_{max}^{SG}\left(t\right)$ the resulting filtered signal.

- The fluctuations of the α−band dominant frequency are measured using the total variation *V*_α_ of the function $\alpha_{max}^{SG}\left(t\right)$ (Figure 2B). This quantity sums the cumulated amplitude of local oscillations in each time intervals and thus measures the deviation of the maximum α−band frequency dynamics with a flat line.

The total variation is computed over a total of M periods of time, where the α−band is present. We apply the sum of the absolute difference between two consecutive frequency time points over the M time segments. For the *k*^*th*^ time segment, we use the local maxima and minima time discretization $\left(t_{0}^{\left(k\right)},..t_{n}^{\left(k\right)}\right)$, (see Figure 2B) so that


(21) $$V_{\alpha }=\frac{1}{\sum_{k=1}^{M}t_{n}^{\left(k\right)}-t_{0}^{\left(k\right)}}\sum_{k=1}^{M}\sum_{i=0}^{n-1}|\alpha_{max}(t_{i+1}^{\left(k\right)})-\alpha_{max}(t_{i}^{\left(k\right)})|.$$

- The δ−band total variation *V*_δ_ is computed with the same computational steps as the α−band with the maximum power frequency δ_*max*_ defined by

(22) $$\begin{array}{l} \delta_{max}\left(t\right)=f_{\delta }\left(t\right)\cdot 1_{\bar{\Omega_{IES}}}\left(t\right), \end{array}$$

where


(23) $$\begin{array}{l} f_{\delta }\left(t\right)=\underset{f\in \left[0.1,4\right]}{arg max}P_{av}\left(f,t\right). \end{array}$$

We compare the spectral properties of the signal before and after filtering (Figure 2C).

### 2.5. Pearson linear correlation coefficient and Bayes factors

The Pearson correlation coefficient *r*_*xy*_ is used to evaluate the correlation between two variables, *x* = (*x*_1_,.., *x*_*n*_) and *y* = (*y*_1_,.., *y*_*n*_), in a sample of *n* points, defined by


(24) $$r_{xy}=\frac{\sum_{k=1}^{n}\left(x-\bar{x}\right)(y_{i}-\bar{y})}{{\left(\sum_{k=1}^{n}{\left(x-\bar{x}\right)}^{2}\right)}^{1/2}{\left(\sum_{k=1}^{n}{\left(y-\bar{y}\right)}^{2}\right)}^{1/2}},$$

where the sample mean is $\bar{x}=\frac{1}{n}\overset{n}{\underset{k=1}{\sum }}x_{i}$ with similar definition for $\bar{y}$.

The null hypothesis *H*_0_ is defined as no correlation between *x* and *y*, while *H*_1_ hypothesis postulate that a correlation exists (29). To provide evidences in favor of the null hypothesis, we use the Bayes factor *BF*_01_ by evaluating the ratio of the conditional probability of the observable *y* given the hypothesis *H*_0_ given by


(25) $$\begin{array}{lll} BF_{10} & = & \frac{P\left(y|H_{1}\right)}{P\left(y|H_{0}\right)}. \end{array}$$

We shall now rewrite each hypothesis in terms of linear regression by considering


(26) $$H_{0}:y=a+\epsilon \text{ and}$$

(27) $$H_{1}:y=a+bx+\epsilon ,$$

where (α, β) are the regression estimates and ϵ~*N*(0, σ^2^*I*), where the constant σ is fixed. The marginal probabilities can be written


(28) $$P(y|H_{\gamma })=\int_{\theta_{\gamma }}P(y|\theta_{\gamma },H_{\gamma })P(\theta_{\gamma }|H_{\gamma })\text{d}\theta_{\gamma }$$

Where γ = 0 or 1 and θ_γ_ are the model parameters (*a, b*, σ^2^). For we add a scalar *g* in the model parameters. When γ = 1, we have


(29) $$\begin{array}{l} y|\theta_{1},H_{1}=y|a,b,\sigma^{2},x\\ \;\sim N(a+bx,\sigma^{2}I), \end{array}$$

and


(30) $$\begin{array}{l} \theta_{1}|H_{1}=a,b,\sigma^{2},g|x\\ \;=(b|a,\sigma^{2},g,x)*(a,\sigma^{2}|g)*(g). \end{array}$$

Thus the probability is given by


(31) $$\begin{array}{l} P\left(\theta_{1}|H_{1}\right)=P\left(b|a,\sigma^{2},g,x\right)P\left(a,\sigma^{2}|g\right)P\left(g\right). \end{array}$$

We follow the steps described in Liang et al. (30) based on Zellner g-priors to get an expression for these probabilities


(32) $$b|g,\sigma^{2},x\;\sim N(0,g\sigma^{2}{\left(x^{T}x\right)}^{-1}),$$

(33) $$P(a,\sigma^{2})=\frac{1}{\sigma^{2}},$$

(34) $$P(g)\;=\;\frac{{\left(n/2\right)}^{1/2}}{\Gamma (1/2)}g^{-3/2}e^{-n/(2g)}.$$

After integrating out all the parameters, we obtain the analytical expression for the Bayes factor


(35) $$\begin{array}{l} BF_{10}=\frac{{\left(n/2\right)}^{1/2}}{\Gamma \left(1/2\right)}{\int_{0}^{\infty }\left(1+g\right)}^{\left(n-p-1\right)/2}\\ \;{\left(1+\left(1-r_{xy}^{2}\right)g\right)}^{-\left(n-1\right)/2}g^{-3/2}e^{n/\left(2g\right)}dg, \end{array}$$

which depends on *p* is the number of covariates in *H*_1_ (30–32).

### 2.6. Partial correlation

The partial correlation coefficient *r*_*xy*∣*z*_ measures the correlation between the data *x* and *y* while controlling for *z*. It is defined by


(36) $$\begin{array}{l} r_{xy|z}=\frac{r_{xy}-r_{xz}r_{yz}}{{\left(1-r_{xz}^{2}\right)}^{1/2}{\left(1-r_{yz}^{2}\right)}^{1/2}}. \end{array}$$

The corresponding Bayes factor compares the models


(37) $$H_{0}:y=a+bx+\epsilon$$

(38) $$H_{1}:y=a+bx+b^{\prime }z+\epsilon .$$

The associated Bayes factor is given by Liang et al. (30), Wetzels and Wagenmakers (31) and Rouder et al. (32)


(39) $$BF_{10}\;=\;\frac{\int_{0}^{\infty }{\left(1+g\right)}^{(n-p^{\prime }-1)/2}{\left(1+(1-R_{1}^{2})g\right)}^{-(n-1)/2}\pi (g)\text{d}g}{\int_{0}^{\infty }{\left(1+g\right)}^{(n-p-1)/2}{\left(1+(1-R_{0}^{2})g\right)}^{-(n-1)/2}\pi (g)\text{d}g},$$

where (*R*_0_, *R*_1_) are the coefficients of determination in (*H*_0_, *H*_1_), (*p, p*′) are the number of covariates in (*H*_0_, *H*_1_).

## 3. Results

To search for possible causal correlation in the EEG between the induction, maintenance phases and the recovery phase of anesthesia, we shall apply the indices presented in the Section 2. We start by estimating the time and frequency shifts of the α−band trajectory during recovery and use them to explore the possible correlations with the total time spent in suppression (iso-electric EEG), a marker of brain sensitivity (5). We will also consider the possible correlation of the frequency shift with respect to the longest suppression induced by a propofol bolus during induction. Finally, we will assess the possible correlation using the total variation, which measures the variation of the band frequency maximum amplitude over time.

### 3.1. Duration and frequency shifts during the α−band emergence trajectory

The EEG signal in the maintenance phase are dominated by two frequency bands: δ−band (0.1–4 Hz) and α−band (8–12 Hz) as depicted in Figures 1A–C. Once the hypnotic injection is stopped, the first step of the recovery phase is characterized by a successive decay of the delta and alpha activity decay. To quantify the time and spectral changes occurring in the EEG during the recovery phase, we shall detect two reference time points *t*_*in*_ and *t*_*out*_, associated with the maximum power frequency within the α−band. The first time *t*_*in*_ is the first instant where the δ−band disappears from the continuous steady-state regime of the maintenance phase. Thereafter, the time *t*_*out*_ corresponds to the instant where the α−band power reaches a lower plateau threshold value.

We identify *t*_*in*_ by applying an optimal fitting procedure of the power *P*_δ_ (Equation 2) with a sigmoid curve *S*_δ_ (Equation 4) and we deduce the first time where *S*_δ_ has reached the threshold λ_*in*_ = 95% of the transient trajectory (see Section 2 and Figure 1D).

Similarly, we fitted the power *P*_α_ (Equation 3) with a sigmoid curve *S*_α_ (Equation 4) and defined the time *t*_*out*_ as the first instant where *S*_α_ has reached λ_*out*_ = 5% of the transient trajectory left to cross (Figure 1E).

Finally, we use this time identification to deduce the duration shift of the α−band emergence trajectory for two population cohorts: for the children cohort, we found the duration is given by Δ*t*_*ROC*_ = 7.8[4.2, 10.2] (Median [IQR]) min and the associated frequency shift is given by Δ*f*_*ROC*_ = 3.3[1.8, 5.0] Hz. For the adult cohort, we found Δ*t*_*ROC*_ = 7.3[5.8, 10.4] min and Δ*f*_*ROC*_ = 1.9[1.3, 2.5] Hz. Interestingly, the distribution of duration Δ*t*_*ROC*_ is similar for both adults and children (Table 1).

**Table 1:** Patients demographic information.

**Cohort**	**Children**	**Adults**
Number	50	27
Age (median [IQR])	5 [4–9] yr	37 [28–48] yr
Age (range)	2–15 yr	16–58 yr
Gender (female/male)	48%/52%	67%/36%
Weight (median [IQR])	20 [16.9–32] kg	85.5 [68–93] kg

### 3.2. Stability of the α−band measured by the total variation

**Figure 3:**
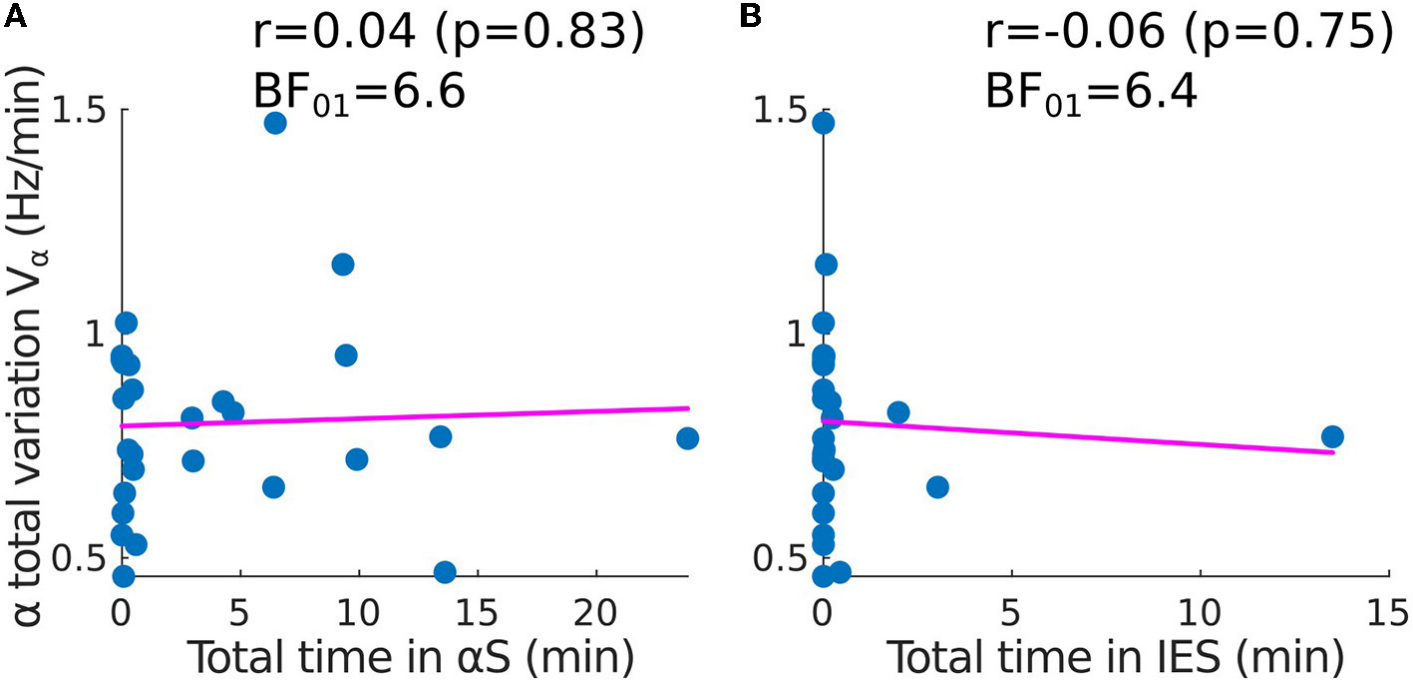
Correlation between the time spent in suppressions and the total variation of the α−band (adult population). Scatter plots and correlations (Pearson *r*, *p*-value *p*) between the α−band total variation *V*_α_ and **(A)** the total time spent in α−suppressions (frequency loss in the α−band), **(B)** the total time spent in IES *S*_*IES*_.

To quantify the stability of the α−band, we estimated how it varies vary from a straight line by using the total variation, a quantity that accounts for any form of oscillation. However, a correlation analysis revealed that the total variation of the α−band is independent of the presence of any suppression patterns such as IES or αS. Indeed, the time spent in suppressions is uncorrelated to the α−band total variation (Figures 3A, B). This result suggests that the IES and total variation can be treated as independent variables in the EEG analysis. The correlation coefficients for the α−suppression (resp. IES) were *r* = 0.04, *p* = 0.83 and the Bayesian factor *BF*_01_ = 6.6 (resp. *r* = −0.06, *p* = 0.75, *BF*_01_ = 6.4). We thus reject with moderate evidence the hypothesis that high oscillations of the α−band would be correlated to αS.

### 3.3. α−band and IES during induction and maintenance are not correlated with α−band emergence trajectory

#### 3.3.1. Statistical correlations for the children cohort

**Figure 4:**
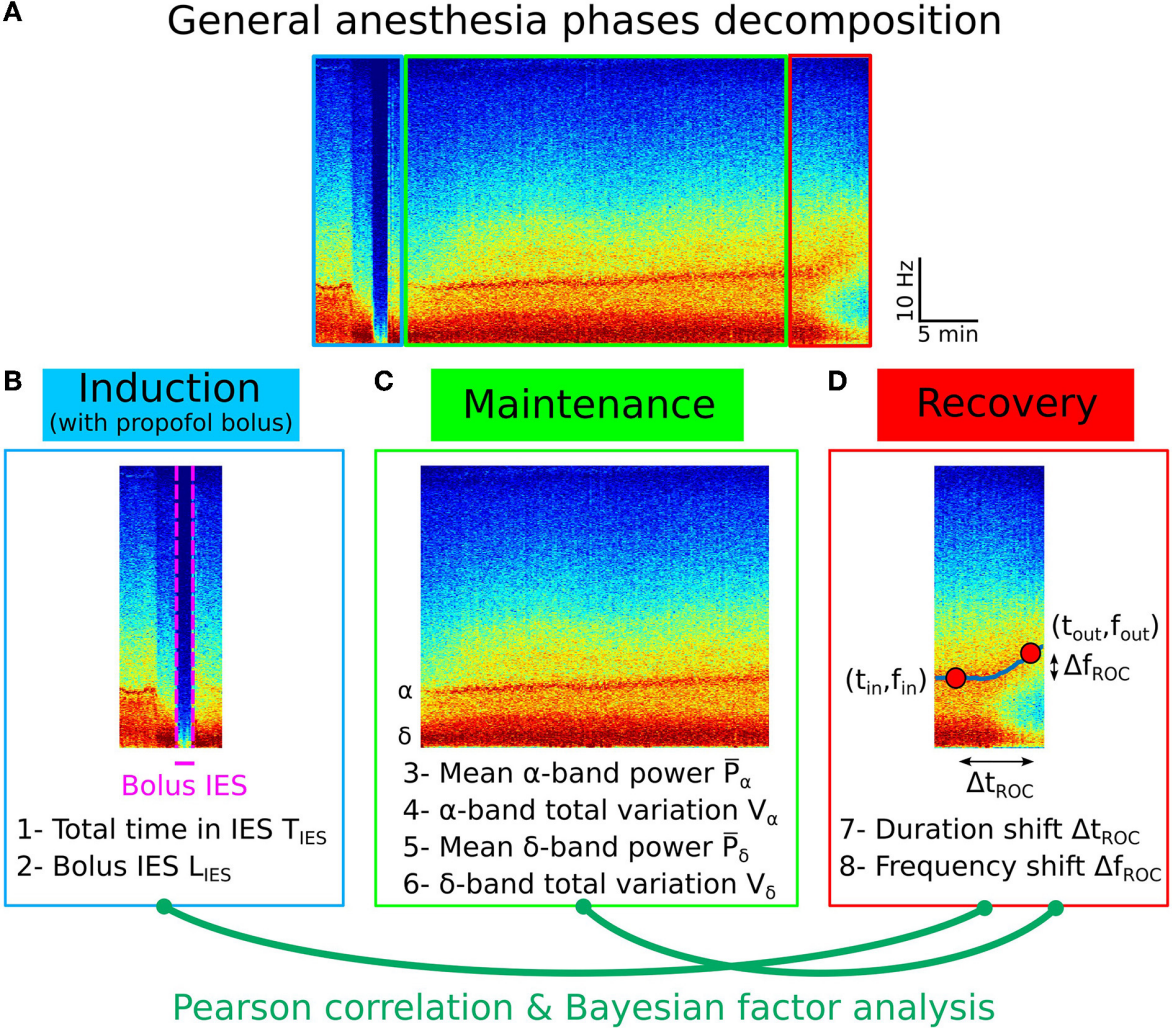
Pearson correlation analysis of the three phases of general anesthesia. **(A)** Spectrogram (time-frequency) representation of the EEG signal during the three phases: induction (blue), maintenance (green), recovery (red). **(B)** Statistical parameters collected during induction: 1- Total time in IES *S*_*IES*_, 2- Longest IES duration *L*_*IES*_. **(C)** Statistical parameters collected during maintenance: 3- Mean α−band power ${\bar{P}}_{\alpha }$, 4- α−band total variation *V*_α_, 5- Mean δ−band power ${\bar{P}}_{\delta }$, 6- δ−band total variation *V*_δ_. **(D)** Statistical parameters collected during recovery: 7- Duration shift Δ*t*_*ROC*_, 8- Frequency shift Δ*f*_*ROC*_.

To determine whether the statistical properties of the recovery phase can be anticipated, we first examined possible correlations from the different variables that we extracted and in particular from the IES for children. We studied the total time spent in IES during maintenance and the IES duration induced by a strong propofol bolus at the end of induction (Figures 4A–D).

We found that (see Section 2.4) the total time spent in IES *S*_*IES*_ = 1.2[0.4, 1.9] min and the longest time spent in IES (induced by propofol bolus) *L*_*IES*_ = 0.3[0.1, 0.7] min.

**Figure 5:**
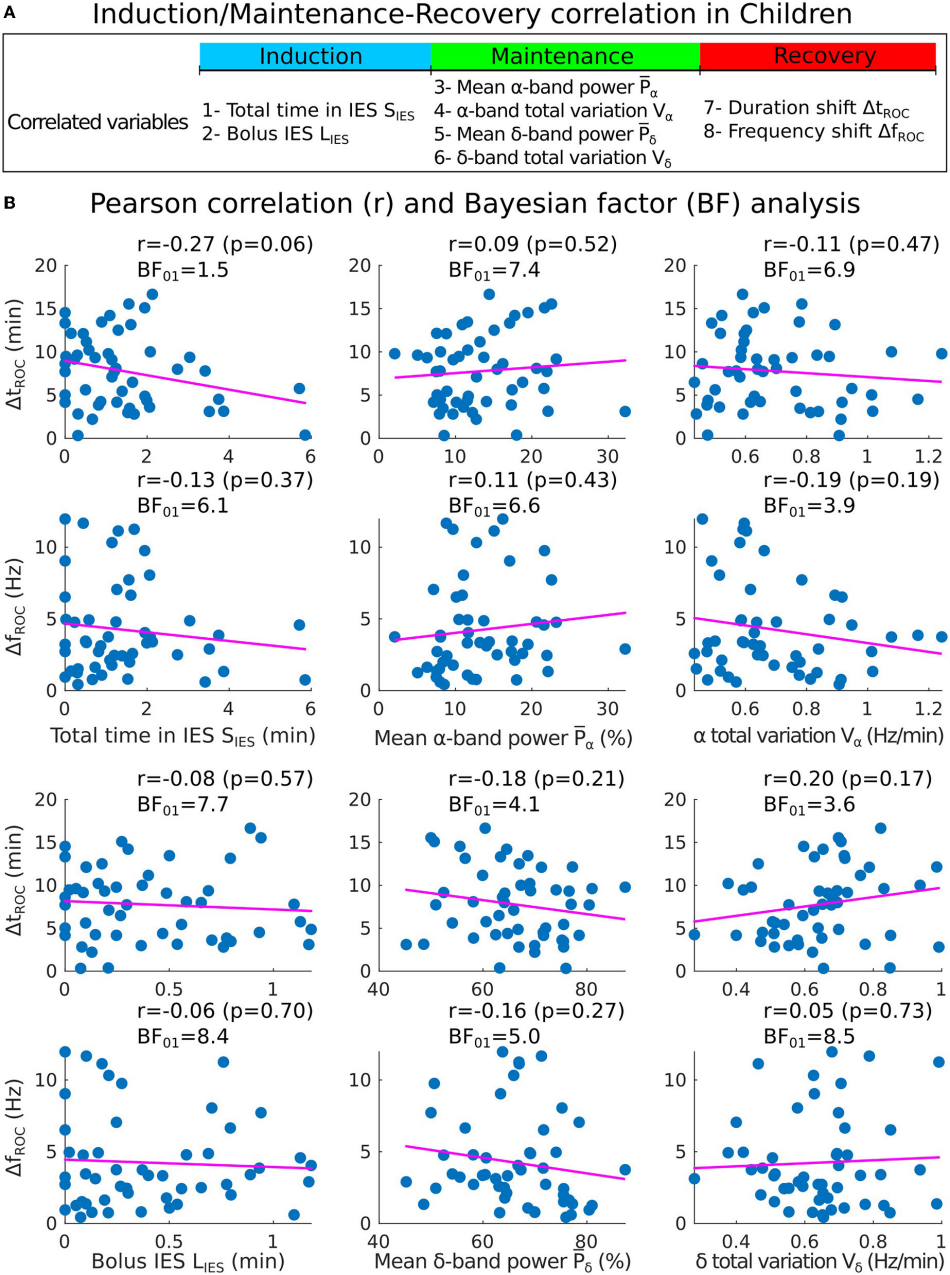
Correlations analysis of induction and maintenance with recovery for 50 children. **(A)** Summary of the statistical parameters collected from the induction and maintenance phases (total time in IES *S*_*IES*_, bolus IES duration *L*_*IES*_, mean α−band power ${\bar{P}}_{\alpha }$ and α−band total variation *V*_α_) and the recovery phase markers (duration shift Δ*t*_*ROC*_ and frequency shift Δ*f*_*ROC*_). **(B)** Scatter plots and Pearson (coefficient *r*, *p*-value *p*, Bayes factor *BF*_01_) correlations between Δ*t*_*ROC*_ (Δ*f*_*ROC*_) and *S*_*IES*_, *L*_*IES*_ (first column), ${\bar{P}}_{\alpha }$, ${\bar{P}}_{\alpha }$ (second column), *V*_α_, *V*_δ_ (third column).

Secondly, we quantified the Pearson correlation (Figure 5A) along with its *p*-value and found that the total duration of the IES (resp. longest IES) is not correlated neither to the duration of recovery Δ*t*_*ROC*_ [*r* = −0.27, *p* = 0.06 (resp. *r* = −0.08, *p* = 0.57)] nor to the frequency shift Δ*f*_*ROC*_ [*r* = −0.13, *p* = 0.37 (resp. *r* = −0.06, *p* = 0.70)], as shown in Figure 5. At this stage, we conclude that no significant correlations are revealed by the transient parameters associated with the electrical absence of the EEG signal.

To further study the possible correlations, we computed the mean power ${\bar{P}}_{\alpha }$ of the α−band during the maintenance and the total variation *V*_α_ which measures the deviation of the maximum frequency *f*_α_ of the α−oscillations from a flat curve (Figures 4C, D). We reported that the mean power ${\bar{P}}_{\alpha }=11.7\left[8.4,17.4\right]\%$ and *V*_α_ = 0.64[0.56, 0.80] Hz/min. We estimated the correlations between ${\bar{P}}_{\alpha }$ (resp. *V*_α_) and either Δ*t*_*ROC*_ [*r* = 0.09, *p* = 0.53 (resp. *r* = −0.09, *p* = 0.53)] or Δ*f*_*ROC*_ [*r* = 0.11, *p* = 0.43 (resp. *r* = −0.16, *p* = 0.27)], but found insignificant outcome.

We repeated the procedure with the same parameters derived from the δ−band and obtained ${\bar{P}}_{\delta }=66.5\left[60.3,74.0\right]\%$ and *V*_δ_ = 0.65[0.54, 0.72] Hz/min. Similarly, the correlations between ${\bar{P}}_{\delta }$ (resp. *V*_δ_) and either Δ*t*_*ROC*_ (*r* = −0.18, *p* = 0.21 (resp. *r* = 0.20, *p* = 0.17)) or Δ*f*_*ROC*_ (*r* = −0.16, *p* = 0.2 (resp. *r* = 0.05, *p* = 0.73) remained non-significant.

However, in our current state, we only failed to reject the null hypothesis of no correlation when obtaining a *p*-value greater than the 5% acceptance level. To support the null hypothesis *H*_0_, we computed the associated Bayes factor (30, 31, 33) *BF*_01_ which is the ratio of the probability between the null hypothesis and the alternative one *H*_1_ considering our data points. According to Jeffreys evidence category scheme (29) for the Bayes factor, our data mostly shows moderate evidence for *H*_0_ as shown in Table 2. We found an anecdotal evidence for *H*_0_ between the total time spent in IES *S*_*IES*_ and the duration shift Δ*t*_*ROC*_. To conclude, the statistics associated with the presence or absence of the α−band do not show any significant correlation with the frequency and duration shifts of the α−band emergence dynamics. In particular, an unstable α−band does not necessarily lead to a longer time of return to consciousness.

**Table 2:** Medians, inter-quartile ranges, Pearson correlations, and Bayes factor of EEG statistical features.

**Measure**	**Median**	**Q1**	**Q3**	**Pearson** *r*		**Bayes Factor** *BF*_01_	
				**1**.	**2**.	**1**.	**2**.
**Children**							
1. Δ*t*_*ROC*_ (min)	7.8	4.2	10.2				
2. Δ*f*_*ROC*_ (Hz)	3.3	1.8	5.0				
3. *S*_*IES*_ (min)	1.2	0.4	1.9	−0.27	−0.13	1.5	6.1^+^
4. *L*_*IES*_ (min)	0.3	0.1	0.7	−0.08	−0.06	7.7^+^	8.4^+^
5. ${\bar{P}}_{\alpha }$ (%)	11.7	8.4	17.4	0.09	0.11	7.4^+^	6.6^+^
6. *V*_α_ (Hz/min)	0.64	0.56	0.8	−0.11	−0.19	6.9^+^	3.9^+^
7. ${\bar{P}}_{\delta }$ (%)	66.5	60.3	74.0	−0.18	−0.16	4.1^+^	5.0^+^
8. *V*_δ_ (Hz/min)	0.65	0.54	0.72	0.20	0.05	3.6^+^	8.5^+^
**Adults**							
1. Δ*t*_*ROC*_	7.3	5.8	10.4				
2. Δ*f*_*ROC*_	1.9	1.3	2.5				
3. *S*_*IES*_	0.1	0	0.6	−0.09	0.03	6.1^+^	6.7^+^
4. *L*_*IES*_	0.03	0	0.09	−0.12	−0.05	5.6^+^	6.5^+^
5. ${\bar{P}}_{\alpha }$	11.4	9.0	16.1	−0.11	−0.05	5.8^+^	6.6^+^
6. *V*_α_	0.8	0.7	0.9	0.11	−0.14	5.8^+^	5.4^+^
7. ${\bar{P}}_{\delta }$	67.7	61.7	75.8	0.03	0.10	6.7^+^	5.9^+^
8. *V*_δ_	0.33	0.27	0.46	−0.44^*^	0.02	0.5	6.7^+^

^*^p < 0.05, ^+^*BF* = 3 − 10.

#### 3.3.2. Statistical correlations in the adults cohort

**Figure 6:**
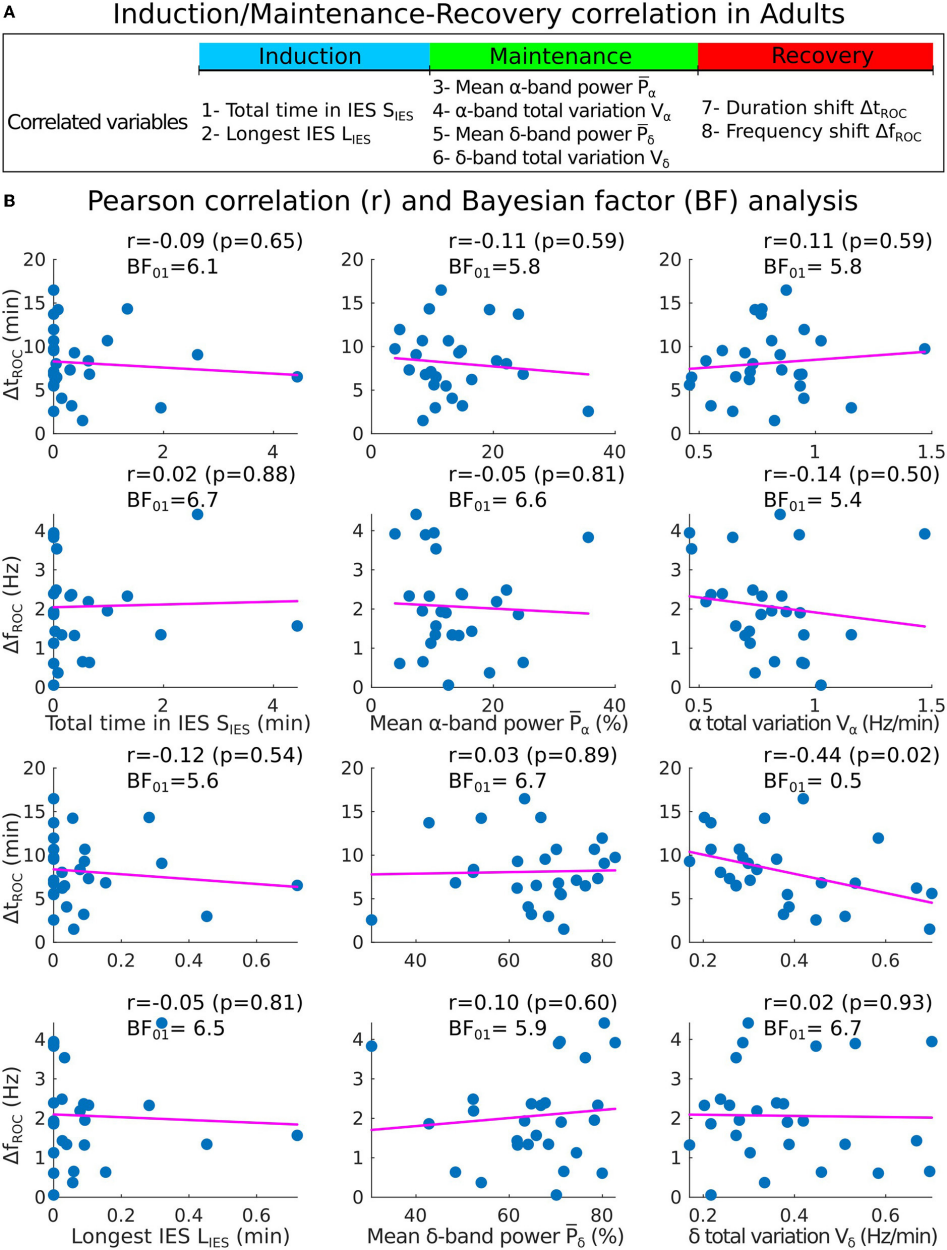
Correlations analysis of induction and maintenance with recovery for 27 adults. **(A)** Summary of the statistical parameters collected during induction and maintenance phases (*S*_*IES*_, *L*_*IES*_, ${\bar{P}}_{\alpha }$ and *V*_α_) and the recovery phase (Δ*t*_*ROC*_ and Δ*f*_*ROC*_). **(B)** Scatter plots and Pearson (coefficient *r*, *p*-value *p*, Bayes factor *BF*_01_) correlations between Δ*t*_*ROC*_ (Δ*f*_*ROC*_) and *S*_*IES*_, *L*_*IES*_ (first column), ${\bar{P}}_{\alpha }$, ${\bar{P}}_{\alpha }$ (second column), *V*_α_ and *V*_δ_ (third column).

We applied the same procedure for adults sedated with propofol target controlled infusion (TCI) protocol. Having, *S*_*IES*_ = 0.1[0, 0.6] min and *L*_*IES*_ = 0.03[0, 0.09] min, we report with moderate evidence that the total time in IES (resp. longest IES) was not correlated neither to the duration shift Δ*t*_*ROC*_ [*r* = −0.09, *p* = 0.65, *BF*_01_ = 6.1 (resp. *r* = −0.12, *p* = 0.54, *BF*_01_ = 5.6)] nor the frequency shift Δ*f*_*ROC*_ [*r* = 0.02, *p* = 0.88, *BF*_01_ = 6.7 (resp. *r* = −0.05, *p* = 0.81, *BF*_01_ = 6.5)] (Figures 6A, B).

We also report the values for the mean power ${\bar{P}}_{\alpha }=11.4\left[9.0,16.1\right]\%$, *V*_α_ = 0.8[0.7, 0.9] Hz/min, ${\bar{P}}_{\delta }=67.7\left[61.7,75.8\right]\%$ and *V*_δ_ = 0.33[0.27, 0.46] Hz/min. We found again moderate evidence of null correlations between the power ${\bar{P}}_{\alpha }$ (resp. *V*_α_) and neither Δ*t*_*ROC*_ [*r* = −0.11, *p* = 0.59, *BF*_01_ = 5.8 (resp. *r* = 0.11, *p* = 0.59, *BF*_01_ = 5.8)] nor Δ*f*_*ROC*_ [*r* = −0.05, *p* = 0.81, *BF*_01_ = 6.6 (resp. *r* = −0.14, *p* = 0.50, *BF*_01_ = 5.4)].

However, for the δ−band parameters, the correlation between *V*_δ_ and Δ*t*_*ROC*_ was significant (*r* = −0.44, *p* = 0.02, *BF*_01_ = 0.5) but the evidence for the alternative hypothesis *H*_1_ was anecdotal. For the remaining parameters, moderate evidence of no significant statistical correlations were found.

We conclude using a statistical approach estimated over two cohorts of adults and children that the duration of the emergence trajectory, characterized by a shift of the maximum α−band is not significantly correlated neither with the fraction of iso-electric suppression nor with the α−band or δ−band dynamics measured by its power and total variation. Thus, these EEG parameters obtained during induction and maintenance of GA cannot be directly used to explain the duration of the α−band emergence trajectory.

#### 3.3.3. Demographic factors provide insignificant role in the correlations analysis

To account for the impact of demographic factors, the correlations of our features were computed while controlling for age, gender, and weight as covariates. By doing so, we eliminated the effect of confounding covariates that could be numerically related to our features of interest. The partial correlation coefficients and their Bayes factors (see Section 2) are shown in Table 3. A significant partial correlation (*r* = −0.29, *p* < 0.05) was found between the total time spent in IES and the duration shift for the children cohort, though the Bayes factor *BF*_01_ = 475 suggests decisive evidence for *H*_0_. In the adult cohort, a moderate but non-significant correlation (*r* = −0.37, *p* = 0.07) between the δ total variation and the duration shift was found, with decisive evidence for *H*_0_ (*BF*_01_ = 257). For the remaining parameters, the Bayes factors support at least very strongly the null hypothesis *H*_0_.

**Table 3:** Partial correlations and Bayes factor of EEG statistical features.

**Measure**	**Pearson** *r*		**Bayes Factor** *BF*_01_	
	**1**.	**2**.	**1**.	**2**.
**Children**				
1. Δ*t*_*ROC*_ (min)				
2. Δ*f*_*ROC*_ (Hz)				
3. *S*_*IES*_ (min)	−0.29^*^	−0.18	475^2^	123^2^
4. *L*_*IES*_ (min)	−0.10	−0.05	124^2^	101^2^
5. ${\bar{P}}_{\alpha }$ (%)	0.08	0.07	130^2^	126^2^
6. *V*_α_ (Hz/min)	−0.05	−0.13	140^2^	202^2^
7. ${\bar{P}}_{\delta }$ (%)	−0.17	−0.12	222^2^	162^2^
8. *V*_δ_ (Hz/min)	0.20	0.08	244^2^	97^1^
**Adults**				
1. Δ*t*_*ROC*_				
2. Δ*f*_*ROC*_				
3. *S*_*IES*_	−0.10	−0.01	44^1^	41^1^
4. *L*_*IES*_	−0.12	−0.13	47^1^	47^1^
5. ${\bar{P}}_{\alpha }$	−0.10	−0.02	46^1^	44^1^
6. *V*_α_	0.10	−0.13	44^1^	43^1^
7. ${\bar{P}}_{\delta }$	−0.01	0.13	40^1^	55^1^
8. *V*_δ_	−0.37	−0.04	257^2^	47^1^

^*^p < 0.05, ^1^*BF* = 30 − 100, ^2^*BF*>100.

### 3.4. Clinical recovery duration are uncorrelated with the IES and bands parameters

**Figure 7:**
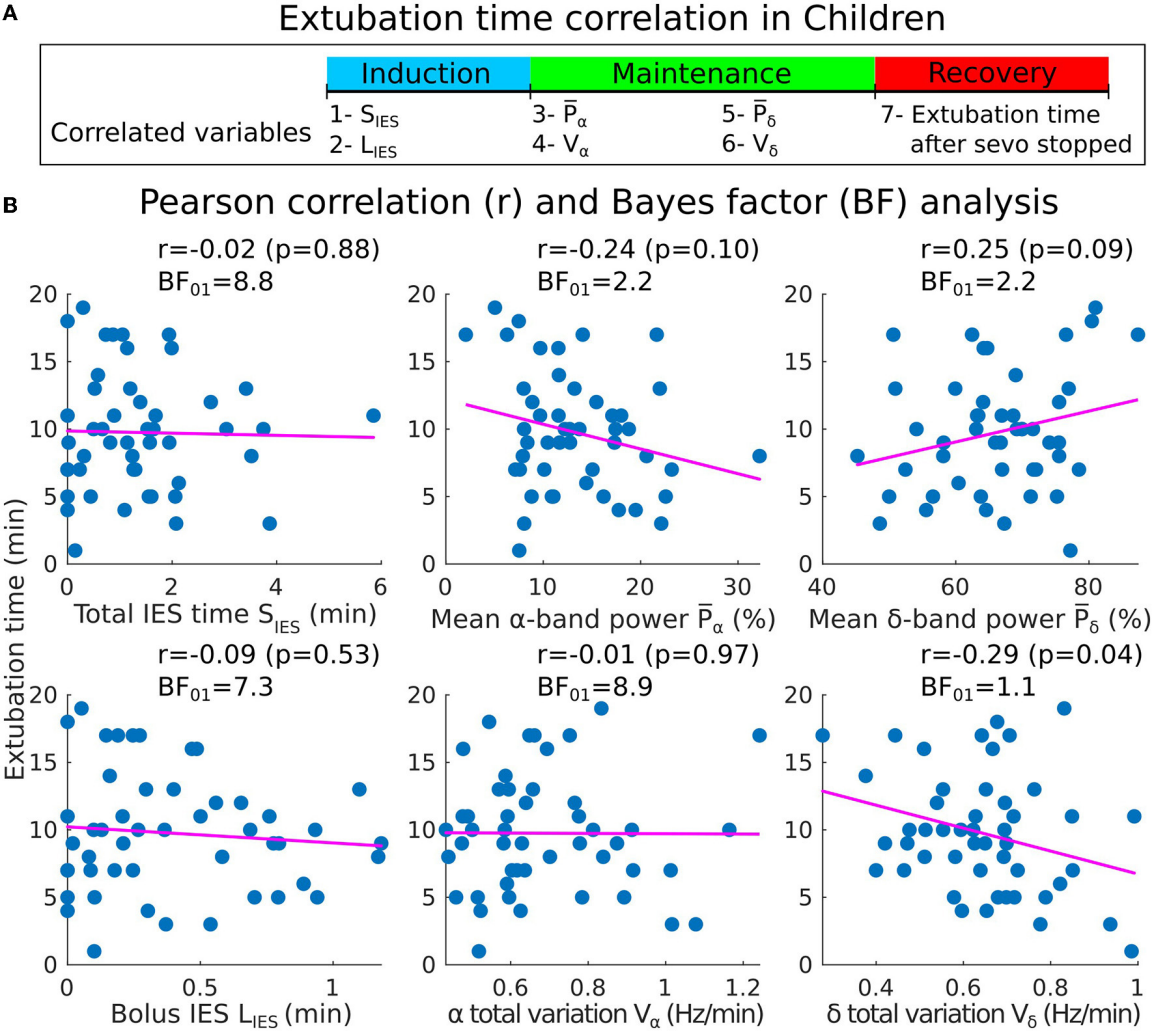
Pearson correlations between the induction/maintenance phases and the extubation time after the sevoflurane has stopped (children cohort). **(A)** Extubation time after the administration of sevoflurane has stopped vs. *S*_*IES*_, *L*_*IES*_, ${\bar{P}}_{\alpha }$ and *V*_α_ collected during induction and maintenance. **(B)** Scatter plots and Pearson correlations (coefficient *r*, *p*-value *p*) between the extubation time and *S*_*IES*_, *L*_*IES*_ (first column), ${\bar{P}}_{\alpha }$, *V*_α_ (second column), ${\bar{P}}_{\delta }$ and *V*_δ_ (third column).

To compare with the results described in the above subsections, we defined the clinical recovery duration from the instant where the hypnotic injection is turned off to the extubation time, where the patient showed the first signs of awareness or recovery of effective spontaneous ventilation. We report with moderate evidence, no correlations between IES total (resp. longest bolus) duration and the extubation time [*r* = −0.02, *p* = 0.88, *BF*_01_ = 8.8 (resp. *r* = −0.09, *p* = 0.53, *BF*_01_ = 7.3)] (Figures 7A, B). Additionally, a moderate evidence of insignificant correlation for the α−band total variation was found (*r* = −0.01, *p* = 0.97, *BF*_01_ = 8.9). For the remaining band parameters, only anecdotal evidences for the null hypothesis were found despite the moderate correlation obtained (${\bar{P}}_{\alpha }$: *r* = −0.24, *p* = 0.10, *BF*_01_ = 2.2; ${\bar{P}}_{\delta }$: *r* = 0.25, *p* = 0.09, *BF*_01_ = 2.2; *V*_δ_: *r* = −0.29, *p* = 0.04, *BF*_01_ = 1.1).

### 3.5. Bayesian inferences shows that longer emergence duration is unlikely to follow long IES duration

To investigate whether a long cumulated IES duration could predict a long α−band emergence duration, we computed the associated empirical conditional probability *Pr*{*t*_*ROC*_>*T*_*ROC*_∣*S*_*IES*_>*T*_*IES*_}. Using the distribution shown in Table 2, we set a threshold at the 70^*th*^ percentile, resulting in *T*_*ROC*_ = 10s and *T*_*IES*_ = 1.8 s for the children cohort. We found that *P*(*t*_*ROC*_>*T*_*ROC*_∣*S*_*IES*_>*T*_*IES*_) = 0.21, while for the adult population, the appropriate threshold changed to *T*_*IES*_ = 0.5 s and the conditional probability was 0.25.

In the children population, we further computed the conditional probability of a long cumulative IES duration when a long IES duration was caused by a propofol bolus. Setting a threshold *T*_*BOL*_ = 0.5 for deciding of the population associated with a long bolus suppression, we found the *P*{*S*_*IES*_>*T*_*IES*_∣*L*_*IES*_>*T*_*BOL*_} = 0.64. To conclude, a Bayesian conditional approach reveals that longer emergence duration is unlikely to follow long IES duration. However, there is a relatively high probability that a longer cumulative IES duration will follow a long IES bolus duration.

## 4. Discussion

In the present study, we used several mathematical indices to quantify the δ and α−bands and to explore the possible correlations of these parameters computed during induction and maintenance with the recovery phase. During induction, we considered the longest IES (induced by a bolus of propofol in children). While adults underwent intravenous hypnotic injection, induction with children starts with inhalation of sevoflurane followed by a bolus of propofol to avoid oro-tracheal intubation complications (34, 35). During maintenance, we computed the mean power ${\bar{P}}_{\alpha }$ of the α−band and the total variation *V*_α_ (Equation 21), a measure of the α−oscillations maximum amplitude instability. Small values of *V*_α_ are associated with low fluctuations around a flat line. Conversely, the higher *V*_α_, the larger is the deviation. Thus, a high value of *V*_α_ characterizes a global instability during anesthesia. During both the induction and maintenance phases, we estimated the total time spent in IES. Finally, during the recovery phase, we estimated the frequency and duration shifts associated to the α−band emergence trajectory from the EEG time-frequency representation, as reported in previous research (25).

The present analysis revealed in general no relationship between the parameters we introduced (Figures 5, 6) in the different phases. These results invalidate our hypothesis concerning a possible correlation between the longest or total IES duration and a longer duration of the α−band emergence trajectory.

Furthermore, our results indicate that the IES durations are not only unrelated with the α−band emergence trajectory, but also with the clinical recovery duration, starting from the instant the hypnotic injection is turned off to the extubation time (Figure 7), where the patient showed first signs of awareness or recovery of spontaneous ventilation and which does not directly follow the end of the emergence trajectory. These findings are consistent with the results reported in Shortal et al. (36), where IES duration was also found to be uncorrelated with the degree of cognitive impairment after anesthesia, as measured by fitting the decay of expired isoflurane to a single exponential.

In addition to the lack of correlation between IES duration and the α−band emergence trajectory in the EEG time-frequency representation, our results also indicate that the α−band dynamics do not show further correlations as revealed by the total variation (Equation 21) of the α−band maximum frequency α_*max*_. Previous research has suggested that a high total variation could reflect a constant re-adjustment that could be induced by real-time hypnotic concentration variation (37, 38). However, our results, which show insignificant correlation between the time spent in suppressions and the α−band total variation (Figure 3), reject this hypothesis. The total variation *V*_α_ could either increase due to a change in the hypnotic concentration or due to the intrinsic response of the brain to a fixed concentration. In the latter case, these changes could represent an instability of thalamo-cortical neuronal networks to converge to a stable constant α−oscillations, which are considered as a marker of brain synchronization (20).

Moreover, the mean α−band power ${\bar{P}}_{\alpha }$ has been used to monitor the depth of anesthesia and as a possible predictor for the arrival of IES (5, 13). Lower values of the power ${\bar{P}}_{\alpha }$ reflects a vulnerable brain and could have been associated to a longer time of emergence. However, this hypothesis was not confirmed in our analysis (Figure 7) where we found insignificant correlation between the mean power ${\bar{P}}_{\alpha }$ and the extubation time.

Based on these findings, it is plausible to suggest that the recovery of the brain from constant anesthetic injection is a memoryless process. Considering that propofol and sevoflurane, both of which are GABAergic agonists (39), enhance the role of inhibitory neurons, the level of neuronal activity mainly depends on the speed at which the hypnotic is absorbed (induction) or eliminated (recovery) by the brain. Indeed, general anesthesia causes thalamic neurons to intrinsically change states (40). During induction, these neurons typically switch from spiking to bursting, while during recovery the switch is from bursting to fast spiking. This is consistent with the changes seen in EEG, where a decrease in the δ (0.1–4 Hz) power and an increase of β (12–30 Hz) power indicate a return to normal neuronal activity (25). During the maintenance phase, the brain has theoretically acclimated to the hypnotics by displaying steady-state neural dynamics.

## Statistical analysis

In this study, statistical analysis was conducted using MATLAB R2021a software. A significance level of α = 0.05 was used throughout the analysis. Qualitative variables were expressed as percentages and quantitative variables were expressed as the median (Inter-Quartile Range: [IQR]). Correlations between variables were determined using the Pearson (*r*) correlation test. To provide evidence in favor of the null hypothesis, Bayes factor (*BF*_01_) was used. No statistical outliers were found or removed in the correlation analyses.

## General statement about general anesthesia

This study is a prospective observational study that included patients who underwent elective procedures requiring standardized general anesthesia procedure. The EEG data used in the study were collected from Louis-Mourier Hospital in Colombes, France between October 2019 and September 2021, in compliance with the evaluation of clinical practice and included a total of 77 patients (50 children and 27 adults), along with their demographic factors and other clinical information. Patients with incomplete or corrupted data were not included in the study. For adult patients, general anesthesia was induced through the intravenous administration of either sufentanil in iterative bolus doses between 5 and 15 gamma or TCI remifentanil ranging from 2.5 to 6 *ng*.*ml*^−1^ for the opioid agent, and propofol for the hypnotic agent with a TCI ranging from 3 to 10 μ*g*.*ml*^−1^ (19). Patients were then intubated following curarization with rocuronium (0.5–0.7 *mg*.*kg*^−1^).

For children, the hypnotic agent sevoflurane was administered through inhalation with an initial concentration of 6%. Following insertion of the intravenous line, the children received 0.1 μ*g*.*kg*^−1^ of sufentanil or 15 μ*g*.*kg*^−1^ of alfentanil. Propofol was then administered in one or more bolus doses until the appearance of iso-electric suppressions for oro-tracheal intubation. The initial quantity of propofol was calibrated according to the child's weight using a 2 *mg*.*kg*^−1^ rule. The anesthesiologist made adjustments to the concentrations depending on the patient's reaction and the anesthesia phase. Additional drugs were given at the discretion of the anesthesiologist as long as the standard of care was respected. Finally, to prepare for the recovery and extubation phase, the concentration of sevoflurane was typically lowered to values between 0.5 and 2.5% 5 to 10 min before complete shut off.

## Data availability statement

The raw data supporting the conclusions of this article will be made available by the authors, without undue reservation.

## Ethics statement

Ethical review and approval was not required for the study on human participants in accordance with the local legislation and institutional requirements. Written informed consent to participate in this study was provided by the participants and/or the participants' legal guardian/next of kin.

## Author contributions

DL and DH: designed research. CS and DH: analyzed data and wrote the manuscript. DL: collected data and corrected the manuscript. All authors contributed to the article and approved the submitted version.

## References

[1] Worrell GA Jerbi K Kobayashi K Lina JM Zelmann R Le Van Quyen M. Recording and analysis techniques for high-frequency oscillations. Progr Neurobiol. (2012) 98:265–78. 10.1016/j.pneurobio.2012.02.006 22420981 PMC3601379

[2] Jaffard S Meyer Y Ryan RD. Wavelets: Tools for Science and Technology. SIAM. (2001). 10.1137/1.9780898718119

[3] Schomer DL Da Silva FL. Niedermeyer's Electroencephalography: Basic Principles, Clinical Applications, and Related Fields. Lippincott Williams & Wilkins (2012). 10.1093/med/9780190228484.001.0001

[4] Ciuciu P Abry P Rabrait C Wendt H. Log wavelet leaders cumulant based multifractal analysis of EVI fMRI time series: evidence of scaling in ongoing and evoked brain activity. IEEE J Select Top Signal Process. (2008) 2:929–43. 10.1109/JSTSP.2008.2006663

[5] Cartailler J Parutto P Touchard C Vallée F Holcman D. Alpha rhythm collapse predicts iso-electric suppressions during anesthesia. Commun Biol. (2019) 2:1–10. 10.1038/s42003-019-0575-3 31508502 PMC6718680

[6] Daubechies I. Ten lectures on Wavelets. SIAM. (1992). 10.1137/1.9781611970104

[7] Averbuch AZ Neittaanmäki P Zheludev VA. Acoustic detection of moving vehicles. In: Spline and Spline Wavelet Methods With Applications to Signal and Image Processing. Springer (2019). p. 219–39. 10.1007/978-3-319-92123-5_12

[8] Flandrin P Amin M McLaughlin S Torrésani B. Time-frequency analysis and applications. IEEE Signal Process Mag. (2013) 30:19. 10.1109/MSP.2013.2270229

[9] Spinnato J Roubaud M Burle B Torrésani B. Detecting single-trial EEG evoked potential using a wavelet domain linear mixed model: application to error potentials classification. J Neural Eng. (2015) 12:036013. 10.1088/1741-2560/12/3/036013 25973635

[10] Dora M Holcman D. Adaptive single-channel EEG artifact removal with applications to clinical monitoring. IEEE Trans Neural Syst Rehabil Eng. (2022) 30:286–95. 10.1109/TNSRE.2022.3147072 35085086

[11] Dora M Jaffard S Holcman D. The WQN algorithm to adaptively correct artifacts in the EEG signal. Appl Comput Harmonic Anal. (2022) 61:347–56. 10.1016/j.acha.2022.07.007

[12] Hastie T Tibshirani R. Friedman J. H. The Elements of statistical Learning: Data Mining, Inference, and Prediction, Vol. 2. Springer (2009).

[13] Shao YR Kahali P Houle TT Deng H Colvin C Dickerson BC. Low frontal alpha power is associated with the propensity for burst suppression: an electroencephalogram phenotype for a “vulnerable brain”. Anesth Anal. (2020) 131:1529. 10.1213/ANE.0000000000004781 33079876 PMC7553194

[14] Sun C Holcman D. Combining transient statistical markers from the EEG signal to predict brain sensitivity to general anesthesia. Biomed Signal Process Control. (2022) 77:103713. 10.1016/j.bspc.2022.103713

[15] Plummer GS Ibala R Hahm E An J Gitlin J Deng H. Electroencephalogram dynamics during general anesthesia predict the later incidence and duration of burst-suppression during cardiopulmonary bypass. Clin Neurophysiol. (2019) 130:55–60. 10.1016/j.clinph.2018.11.003 30476711 PMC6377070

[16] Kay DC Pickworth WB Neidert GL Falcone D Fishman PM Othmer E. Opioid effects on computer-derived sleep and EEG parameters in nondependent human addicts. Sleep. (1979) 2:175–91. 10.1093/sleep/2.2.175 232563

[17] Smith NT Dec-Silver H Sanford TJ Jr Westover CJ Jr Quinn ML Klein F. EEGs during high-dose fentanyl-, sufentanil-, or morphine-oxygen anesthesia. Anesth Anal. (1984) 63:386–93. 10.1213/00000539-198404000-00002 6230952

[18] Egan TD. Remifentanil pharmacokinetics and pharmacodynamics: a preliminary appraisal. Clin Pharmacokinetics. (1995) 29:80–94. 10.2165/00003088-199529020-00003 7586903

[19] Absalom AR Mason KP. Total Intravenous Anesthesia and Target Controlled Infusions. Springer (2017).

[20] Brown EN Lydic R Schiff ND. General anesthesia, sleep, and coma. N Engl J Med. (2010) 363:2638–50. 10.1056/NEJMra0808281 21190458 PMC3162622

[21] Purdon PL Sampson A Pavone KJ Brown EN. Clinical electroencephalography for anesthesiologistspart I: background and basic signatures. Anesthesiology. (2015) 123:937–60. 10.1097/ALN.0000000000000841 26275092 PMC4573341

[22] Hughes SW Crunelli V. Thalamic mechanisms of EEG alpha rhythms and their pathological implications. Neuroscientist. (2005) 11:357–72. 10.1177/1073858405277450 16061522

[23] Cascella M Bimonte S Muzio MR. Towards a better understanding of anesthesia emergence mechanisms: research and clinical implications. World J Methodol. (2018) 8:9. 10.5662/wjm.v8.i2.9 30345225 PMC6189114

[24] Kim H Moon JY Mashour GA Lee U. Mechanisms of hysteresis in human brain networks during transitions of consciousness and unconsciousness: theoretical principles and empirical evidence. PLoS Comput Biol. (2018) 14:e1006424. 10.1371/journal.pcbi.1006424 30161118 PMC6135517

[25] Purdon PL Pierce ET Mukamel EA Prerau MJ Walsh JL Wong KFK. Electroencephalogram signatures of loss and recovery of consciousness from propofol. Proc Natl Acad Sci USA. (2013) 110:E1142–51. 10.1073/pnas.1221180110 23487781 PMC3607036

[26] Vijayan S Ching S Purdon PL Brown EN Kopell NJ. Thalamocortical mechanisms for the anteriorization of alpha rhythms during propofol-induced unconsciousness. J Neurosci. (2013) 33:11070–5. 10.1523/JNEUROSCI.5670-12.2013 23825412 PMC3718379

[27] Ragnemalm I. Fast erosion and dilation by contour processing and thresholding of distance maps. Pattern Recogn Lett. (1992) 13:161–6. 10.1016/0167-8655(92)90055-5

[28] Savitzky A Golay MJ. Smoothing and differentiation of data by simplified least squares procedures. Anal Chem. (1964) 36:1627–39. 10.1021/ac60214a04721322220

[29] Jeffreys H. The Theory of Probability. OUP Oxford (1998).

[30] Liang F Paulo R Molina G Clyde MA Berger JO. Mixtures of g priors for Bayesian variable selection. J Am Stat Assoc. (2008) 103:410–23. 10.1198/016214507000001337

[31] Wetzels R Wagenmakers EJ. A default Bayesian hypothesis test for correlations and partial correlations. Psychon Bull Rev. (2012) 19:1057–64. 10.3758/s13423-012-0295-x 22798023 PMC3505519

[32] Rouder JN Morey RD Speckman PL Province JM. Default Bayes factors for ANOVA designs. J Math Psychol. (2012) 56:356–74. 10.1016/j.jmp.2012.08.001

[33] Ly A Verhagen J Wagenmakers EJ. Harold Jeffreys's default Bayes factor hypothesis tests: explanation, extension, and application in psychology. J Math Psychol. (2016) 72:19–32. 10.1016/j.jmp.2015.06.004

[34] Asai T Koga K Vaughan R. Respiratory complications associated with tracheal intubation and extubation. Br J Anaesth. (1998) 80:767–75. 10.1093/bja/80.6.767 9771306

[35] Kwak H Kim J Kim Y Chae Y Kim J. The optimum bolus dose of remifentanil to facilitate laryngeal mask airway insertion with a single standard dose of propofol at induction in children. Anaesthesia. (2008) 63:954–8. 10.1111/j.1365-2044.2008.05544.x 18557970

[36] Shortal B Hickman L Mak-McCully R Wang W Brennan C Ung H. Duration of EEG suppression does not predict recovery time or degree of cognitive impairment after general anaesthesia in human volunteers. Br J Anaesth. (2019) 123:206–18. 10.1016/j.bja.2019.03.046 31202561 PMC6676227

[37] Hight D Voss LJ Garcia PS Sleigh J. Changes in alpha frequency and power of the electroencephalogram during volatile-based general anesthesia. Front Syst Neurosci. (2017) 11:36. 10.3389/fnsys.2017.00036 28611600 PMC5446988

[38] Flores FJ Hartnack KE Fath AB Kim SE Wilson MA Brown EN. Thalamocortical synchronization during induction and emergence from propofol-induced unconsciousness. Proc Natl Acad Sci USA. (2017) 114:E6660–8. 10.1073/pnas.1700148114 28743752 PMC5558998

[39] Alkire MT Hudetz AG Tononi G. Consciousness and anesthesia. Science. (2008) 322:876–80. 10.1126/science.1149213 18988836 PMC2743249

[40] Steriade M. Impact of network activities on neuronal properties in corticothalamic systems. J Neurophysiol. (2001) 86:1–39. 10.1152/jn.2001.86.1.1 11431485

